# Differences in the gut microbiomes of dogs and wolves: roles of antibiotics and starch

**DOI:** 10.1186/s12917-021-02815-y

**Published:** 2021-03-06

**Authors:** Yuting Liu, Bo Liu, Chengwu Liu, Yumiao Hu, Chang Liu, Xiaoping Li, Xibao Li, Xiaoshuang Zhang, David M. Irwin, Zhiqiang Wu, Zeliang Chen, Qi Jin, Shuyi Zhang

**Affiliations:** 1 Institute of Pet Sciences, College of Animal Science and Veterinary Medicine, Shenyang Agricultural University, Shenyang, China; 2 Ministry of Health Key Laboratory of Systems Biology of Pathogens, Institute of Pathogen Biology, Chinese Academy of Medical Sciences and Peking Union Medical College, Beijing, China; 3 Shenyang Police Dog Technical College, Shenyang, China; 4 Shenyang Forest Zoological Garden, Shenyang, China; 5 Changchun Animals and Plants Park, Changchun, China; 6 Department of Laboratory Medicine and Pathobiology, University of Toronto, Toronto, ON M5S 1A8 Canada

**Keywords:** Dog, Wolf, Gut bacteria, Antibiotic, Public health

## Abstract

**Background:**

Dogs are domesticated wolves. Change of living environment, such as diet and veterinary care may affect the gut bacterial flora of dogs. The aim of this study was to assess the gut bacterial diversity and function in dogs compared with captive wolves. We surveyed the gut bacterial diversity of 27 domestic dogs, which were fed commercial dog food, and 31 wolves, which were fed uncooked meat, by 16S rRNA sequencing. In addition, we collected fecal samples from 5 dogs and 5 wolves for shotgun metagenomic sequencing to explore changes in the functions of their gut microbiome.

**Results:**

Differences in the abundance of core bacterial genera were observed between dogs and wolves. Together with shotgun metagenomics, the gut microbiome of dogs was found to be enriched in bacteria resistant to clinical drugs (*P* < 0.001), while wolves were enriched in bacteria resistant to antibiotics used in livestock (*P* < 0.001). In addition, a higher abundance of putative α-amylase genes (*P* < 0.05; *P* < 0.01) was observed in the dog samples.

**Conclusions:**

Living environment of dogs and domestic wolves has led to increased numbers of bacteria with antibiotic resistance genes, with exposure to antibiotics through direct and indirect methods. In addition, the living environment of dogs has allowed the adaptation of their microbiota to a starch-rich diet. These observations align with a domestic lifestyle for domestic dogs and captive wolves, which might have consequences for public health.

## Background

Dogs (*Canis lupus familiaris*) were probably the first and only animal domesticated before the advent of settled agriculture [1]. The history of dog domestication is often considered to be a two-stage process, where primitive dogs were first domesticated from gray wolves (*Canis lupus laniger*) and then, in a second stage, further selection on these primitive forms yielded the many specialized dog breeds found today [2–4]. Recent investigations suggest that the novel adaptations allowing early ancestors of dogs to thrive on diets rich in starch, in comparison to the carnivorous diet of wolves, was a crucial step in domestication [5]. Since the typical food source for wolves are ungulates, such as wild boar, and small mammals, the adaptation of dogs to eating grains and other vegetation is reflected in changes in the dog genome to the sequences of genes involved in starch and glucose metabolism [5].

Microbes have been found living in the gut of virtually all metazoans, including both invertebrates and vertebrates [6]. It is commonly appreciated that the activity of microbes, and their metabiotic products, play important roles in the health of mammals, including humans [7, 8]. Adaptation and convergence of microbiota to diet occurs across mammals, and food consumed by a mammal influences its gut microbiota [9]. For example, diets rich in plant fiber promote a gut microbiota that is considerably different from the microbiota found with diets rich in animal fat [10]. It has been shown that the microbial composition of the gut of the giant panda differs from its carnivorous close relatives, likely due to the adaptation of its gut microbiota to the digestion of bamboo [11]. A comparative studies with 51 breeds of dogs has shown that diet influences bacterial composition and function [12]. To date, however, reconstruction of host-microbe evolutionary histories has been limited, and additional studies are needed on the gut microbiomes of wild animals [13]. An area of interest is the diversity of antibiotic resistance in the gut bacteria of dogs. For example, cephalosporin-resistant *Enterobacteriaceae* was found to be prevalent among dogs of various backgrounds living in animal shelters [14]. Similarly, a study of companion animals in North-West Germany found that 2.6% of the dogs in this population possessed methicillin-resistant *Staphylococcus aureus* and 3.6% of them had extended spectrum beta-lactamase-producing *Enterobacteriaceae* [15]. In addition, a study that characterized and compared antibiotic resistance by fecal *E. coli* isolates from dogs and their owners found that the most prevalent resistance gene was against sulfamethoxazole gene [16]. These studies suggest that differences in the prevalence of antibiotic resistant bacteria exist within dogs that might reflect their living conditions.

In this study, we addressed this question by assessing the composition of the gut microbiota found in 27 dogs, belonging to 3 different breeds, and 31 captive wolves, initially using bacterial 16S rRNA sequences from feces samples, followed by the parallel deciphering of microbial genomes from five samples from each population, to assess the functional consequences of the microbes to their hosts. We found that the gut microbes in dogs and wolves possess unique genes involved in antibiotic resistance, which might echo direct and indirect antibiotic intake. In addition, genes related to starch metabolism are found in greater abundance in the gut microbes of dogs compared to wolves, which might assist the better utilization of starch by dogs.

## Results

### Comparative analysis of 16S communities of *Canis lupus*

Gut bacteria in the two groups of animals (27 dogs and 31 wolves) were identified from Illumina 16S ribosomal DNA V4–V5 hyper variable region sequence data from fecal samples. A total of 3,858,805 effective tags were obtained, with an average of 72,808 tags per sample. Tags were clustered into 14,118 operational taxonomic units (OTUs) using a 97% sequence identity cutoff. Rarefaction curves for phylogenetic diversity plateaued, approximating a saturation phase, after 7000 sequence per sample.

**Fig. 1:**
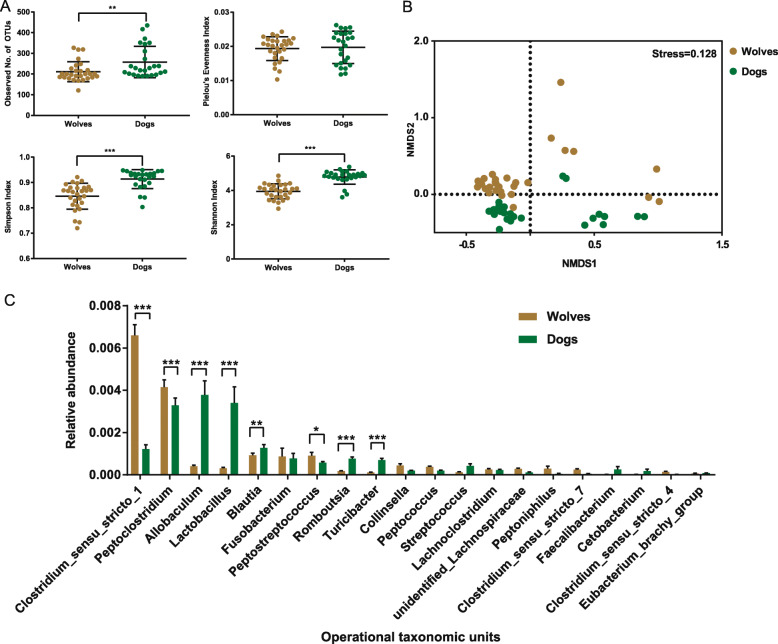
Gut microbial communities in dogs and wolves are different. **a** Variation in microbial diversity and richness in dogs and wolves was calculated using the observed number of OTUs, Shannon index, Simpson index and Pielou’s evenness index. (*P < 0.05; **P < 0.01; ***P < 0.001). **b** NMDS between the gut microbiota from pairs of animals. Each node represents a pair of samples. Note, the gut bacteria of wolves from different zoos cluster together, indicating that the influence of different feeding areas is weak. **c** Histograms of the proportions of top 20 OTUs classified at the genus level. OTUs were compared by the Holm–Sidak method t-test for all OTUs, with significant differences indicated by asterisks (*P < 0.05; **P < 0.01; ***P < 0.001)

The α-diversity of the gut microbes, which was measured using the observed numbers of OTUs (*P* < 0.01), Shannon index (*P* < 0.001) and Simpson index (*P* < 0.001), was significantly higher within the dog group than within wolves (Fig. 1a). We then compared the overall community structure and composition of the microbiota between the two groups. Interestingly, both groups showed highly separated clustering for NMDS (Nursing Minimum Data Set) distances (Fig. 1b). We also found that dogs and wolves have different microbial community compositions, with *Allobaculum* (Kruskal-Wallis; LDA = 4.93, *P* < 0.001) and *Lactobacillus* (Kruskal-Wallis; LDA = 4.91, *P* < 0.001) dominating in dogs, while wolves possess more *Clostridium* sensu stricto *1* (Kruskal-Wallis; LDA = 5.17, *P* < 0.001) (Fig. 1c).

### Antibiotic resistance profiling of the gut microbiomes of dogs and wolves

Shotgun metagenomic data can be used to assess the metabolic repertoire of the entire complex microbial population by analyzing coding genes within the microbiomes [17]. To examine the consequence of the genetic changes of the dog compared to the wolf on the composition of their gut microbiomes, we investigated metagenomes from 5 dogs (ASD04, ASD05, D19, D20, and D23) and 5 wolves (ASW03, ASW04, ASW05, W28, and W27) using shotgun metagenomic sequencing. The 10 samples were selected based on their 16S microbial profiles to minimize intragroup differences, nevertheless, we cannot eliminate difference other than composition. The shotgun metagenomic approach generated a total of 869,723,146 reads with an average of 86,972,315 reads per individual.

**Fig. 2:**
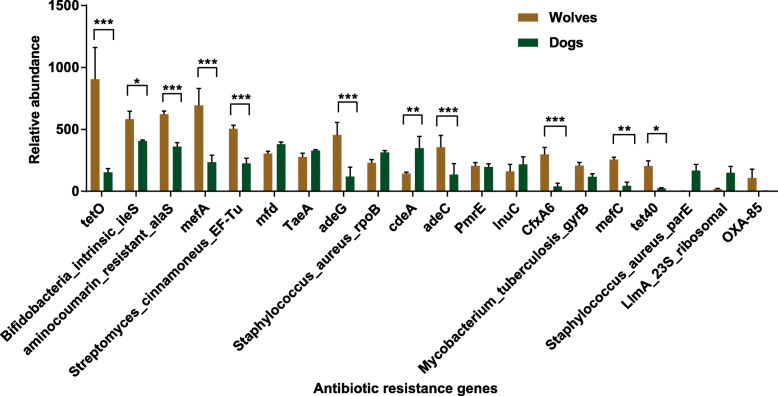
Abundance of different types of antibiotic resistance genes (ARG) in the gut microbiomes of dogs and wolves. Histograms of the relative abundance of top 20 ARGs in each group. Significance of the differences was tested by Holm–Sidak method and indicated by asterisks (*P < 0.05; **P < 0.01; ***P < 0.001)

We searched for antibiotic resistance genes (ARGs) with the CARD database [18–20], where we found that ARGs account for 0.58 and 0.84% of the genes in dog and wolf microbial metagenomes, respectively. More specially, genes coding for ARGs such as *cdeA*, were more abundant in dogs, while *tetO*, *Bifidobacteria intrinsic ileS*, *aminocoumarin resistant alaS*, *mefA*, *Streptomyces cinnamoneus EF-Tu*, *adeG*, *adeC*, *CfxA6*, *mefC* and *tet40* are more prominent in wolves (Fig. 2). In addition, although *Staphylococcus aureus parE* and *LlmA 23S ribosomal* genes were not among the top ten most abundant gene types in dogs, they were about 270 and 12 times more abundant, respectively, in the wolf.

### Putative glycan degrading enzymes characterization of dogs and wolves

**Fig. 3:**
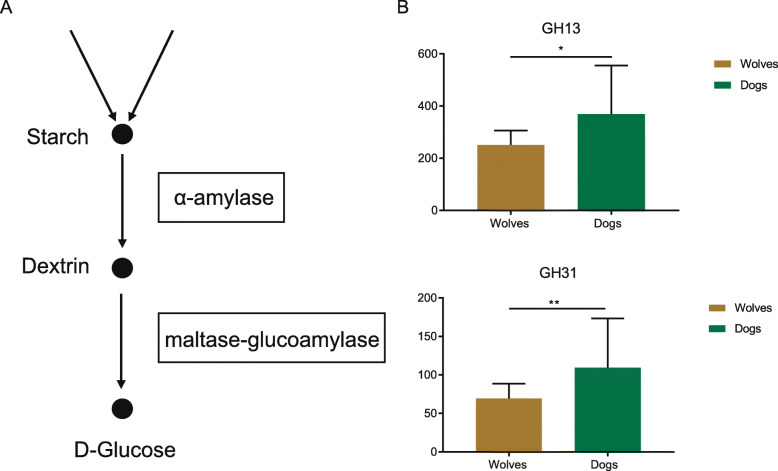
Abundance of genes associated with starch digestion in the metagenomes of the dog and wolf are different. **a** Pathway for starch digestion, from KEGG. **b** Histogram showing the normalized distribution of the abundance of genes encoding enzymes related to starch digestion is significantly enriched in the dog microbiome relative to the wolf. Significant differences are indicated by asterisks (*P < 0.05; **P < 0.01; ***P < 0.001)

The breakdown of starch, as shown in the Kyoto Encyclopedia of Genes and Genomes (KEGG) pathway, proceeds in two stages: (1) starch is first cleaved to yield dextrin by α-amylase, and then (2) dextrin is hydrolyzed by maltase-glucoamylase (MGA) to form D-glucose *(*Fig. 3a). Interesting, putative Glycosyl Hydrolases (GHs) genes related to starch digestion, such as GH13 (α-amylase) and GH31 (MGA), exhibit significantly higher abundance in the dog microbial metagenome compared to wolves (Fig. 3b). In addition, gene families involved in carbohydrates digestion such as GH3 (L-arabinofuranosidase), GH32 (invertase), GH36 (α-galactosidase), GH39 (β-xylosidase), and GH77 (amylomaltase), were also more abundant in the dog microbial metagenome.

## Discussion

Bacterial 16S rRNA sequences were used to assess the composition of the gut microbiota from feces samples of 27 dogs, belonging to 3 different breeds, and 31 captive wolves. Bioinformatic analyses performed to evaluate whether differences in the gut microbiota existed between the canine breeds found no differences. A recent study comparing domestic dog breeds and their wild relatives also have suggested that host phylogeny only plays a minor role in the modulation of gut populations in *Canis lupus* [12]. Therefore, we ignored differences among dogs and considered them as a group. Analyses of alpha-diversity analysis, such as observed number of OTUs, Shannon index, Simpson index and Pielou’s evenness index, show that the microbiota of dogs have a higher diversity than that of wolves, which might be due to the diversity of the dog food.

Examination of the abundance of different species in the microbiota showed that the genus *Clostridium* sensu stricto *1* was most prevalent (35.6%) in the wolf microbiota, which is consistent with the results of Wu et al. [21], suggesting the co-evolution between this genera and the wolf gut. In the dog, the genera *Lactobacillus* (17.5%) and *Allobaculum* (19.1%) were the most prevalent, which might be due to the change in their diet to one that is high in carbohydrates and fiber.

Gut resistome research typically focuses on humans, where numerous and diverse resistance gene orthologues and the origins of drug resistance genes in the clinic have long been debated [22, 23]. Today, veterinary antibiotics (VAs) are widely used in many countries to treat diseases in pets and to improve growth rates and feed efficiency in farming livestock [24]. This has resulted in increased levels of antibiotic resistance in the gut flora of food animals, which subsequently enters the food chain, or goundwater, of other ominvores and carnivores [23, 25], and potentially having deleterious effects [26]. Therefore, a comparison of the gut microbe resistome of the wolf to that of the domestic dog might provide insight into how antibiotic use, both direct and indirect use, has altered antibiotic resistance in the gut microbiome of *Canis lupus*. Furthermore, research on the gut resistome in domestic dogs might provide clues concerning antibiotic resistance in dogs, identifying antibiotics that should suggest be replaced for increased efficacy.

Compared to wolves, the dog gut microbiome is considerable enriched for *cdeA*, *Staphylococcus aureus parE*, and *llmA*, which confer resistance to fluoroquinolone, novobiocin, and clindamycin, respectively. Indeed, enrofloxacin (fluoroquinolones) and clindamycin are common clinical antimicrobial drugs consumed by sick dogs [27] and *cdeA* was found to be enriched in the gut microbiota of infant humans treated with antibiotics [28]. This observation suggested that the fluoroquinolone and clindamycin resistance in dogs likely comes from veterinary drugs, and that dogs have undergone selective evolution due to clinical exposure to antibiotics. Moreover, resitance to some antibiotics, such as novobiocin that is rarely used in the treatment of dogs, potentially was acquired through exposure to these antibiotics from pet food.

Unexpectedly, we found a diverse set of ARGs (*tetO and tet40* conferring resistance to tetracycline; *Bifidobacteria intrinsic ileS* show resistance to mupirocin; *aminocoumarin resistant alaS* show resistance to novobiocin; *mefA* show resistance to macrolide; *Streptomyces cinnamoneus EF-Tu* conferring resistance to elfamycin; *adeG*, *mefC* and *adeC* show multidrug resistance and *CfxA6* conferring resistance to cephamycin) in wolves. Since uncooked meat is the primary food source for the wolves, we suspect that this is the source of these antibiotic resistance genes. Livestock, such as chicken, are treated with antimicrobials during production to maintain health and productivity [29, 30]. Thus, uncooked meat will contain bacteria with antibiotic exposure, which could then be indirectly transferred to a predator (e.g., wolf) via the digestive system. In contrast, high temperature processing in the production of dog food production would lead to the destruction of ARGs and drug-resistant bacteria. It is worth noting that humans also obtain protein via livestock, thus, the long-term consequences of the consumption of under-cooked meat potentially has serious consequences for public health and threaten the sustainability of the livestock industry.

In 2019, Alessandri et al. showed that different diets in dogs resulted in differentiated microbiota, however, with a core set of of gut bacteria genera that did not fluctuate, which might be due to extensive co-evolution with the host [12]. We hypothesize that among the environmental factors separating our two populations (diet, sanitation, hygiene, geography, and climate), the presence of *Allobaculum* could be a consequence of high fiber intake, and maximizing metabolic energy extraction from ingested plant polysaccharides.

It has been reported that *Clotridium* sensu stricto *1* and *Allobaculum* have been linked to protein and lipid degradation [31, 32]. However, *Lactobacillus* can help ferment carbohydrates. Enhanced starch digestion, through AMY2B copy number expansion in the dog genome, has been postulated to be an adaptation to the shift from the carnivorous diets of wolves to the starch-rich diets of the domesticated dog [5]. Our dog microbiome samples show higher abundances of putative GH13 and GH31 type genes compared to the wolf. This result suggests that the increased amylase generated by the changes in the dog genome may not completely explain the shift to the starch-rich diet in dogs, and that changes in the composition of gut microbes might help break down starch-rich food. Gut microbes in the domestic dogs should be better able to digest starch compared to those of captive wolves, thus we hypothesize that the gut microbes from free running wolves that have a significantly greater amount of aerobic exercise and fewer opportunities to eat grain should have a weaker ability to digest starch compared to our captive wolves.

## Conclusions

In summary, our findings demonstrate that long-term domestication has affected the gut microbes of dogs leading to increases in the number of coding genes for starch digestion and antibiotic resistance. Furthermore, direct consumption of uncooked livestock products also indirectly leads to increases in ARGs. Nevertheless, the two groups differ in many other variables such as amount of aerobic exercise, hygiene, exposure to human (for example: veterinary care) thus, it is difficult to disentangle the role of genetic and environmental changes in shaping the domestic microbiome due to captivity.

## Methods

### Sample collection

Fecal samples from 27 adult police dogs (*Canis lupus familiaris*) (including 22 purebred German Shepherds, 4 purebreds Belgian Malinois, and 1 purebred English Springer Spaniel) (25–96 months old) and 31 wolves (*Canis lupus laniger*) (adult) for this project were collected between December 2016 and January 2017. Dogs were from a dog-breeding center where the animals were individually kept in kennels and this dog-breeding center only had these three breeds. Investigators were gloved, masked, and gowned during sampling. To avoid non-physiological changes in the fecal microbiota and contamination with organisms from the environment, fresh feces were collected as soon after defecation as possible, when the fecal material was still warm, soft, and smelly. A sterile medicine spoon was used to remove the outer part of the feces and each sample were transferred into a tube using a new sterile medicine spoon as quickly as possible. Tubes with fecal samples were kept initially on dry ice and then stored at − 80 °C until processing.

Wolf fecal samples were similarly collected from communal pens at two zoos (Shenyang Forest Zoological Garden, Liaoning, China, 17 wolves; Changchun Plant and Animal Park, Jilin, China, 14 wolves). While wild free running wolves might be more suitable for this study, collecting samples from these animals, however, would have been more difficult, thus we collected from captive wolves that are recent (a few generations) descendants of wild-caught wolves. The habits of these wolves show no evidence of domestication.

### Diets and treatments of the dogs and wolves

The diet for the police dogs was composed of commercial dog food, which contains grain (rice/wheat/corn), meat, vitamins, and minerals, and was manufactured by Pedigree, MARS, China. Moreover, as puppies, these dogs were injected with a combination vaccine to prevent viral diseases and the police dog breeding center is equipped with a dog hospital where cephalosporins (β-lactams), gentamicin (aminoglycosides), and enrofloxacin (fluoroquinolones) are commonly used as medication.

Wolves were primarily fed unprocessed chicken carcasses and beef, which were purchased from markets and not labeled as organic, and could eat grass found on the grounds of their habitats. However, live sheep were occasionally put in with the wolves, as evidenced by white bones seen in their habitats. Although zoo animals did give some medical attention, including treatment with antibiotics, this was rare as injured and sick wolves usually healed by themselves, and treatment of them can be extremely dangerous for the breeders. The wolf populations are descendants of wild wolves that were captured a few decades ago, and have since multiplied in a fenced area of three thousand square meters.

### DNA extraction and sequencing

Total bacterial DNA was extracted at Novogene Bioinformatics Technology Co., Ltd. (Beijing, China) using TIANGEN kits according to the manufacturer’s recommendations. Approximately 40–200 mg of fecal material was used for each extraction.

The hypervariable V4-V5 region of the 16S rRNA gene was amplified using specific primers (515F: GTG CCA GCM GCC GCG G; 907R: CCG TCA ATT CMT TTR AGT TT). All PCR reactions were carried out with Phusion® High-Fidelity PCR Master Mix (New England Biolabs). The same volume of 1X loading buffer (containing SYB green) was mixed with PCR products, which were then separated by electrophoresis on 2% agarose gels. Bright bands between 400 and 450 bp in length were chosen for further analysis. Selected PCR product bands were then mixed in equidense ratios and purified with the Qiagen Gel Extraction Kit (Qiagen, Germany). Sequencing libraries were generated using TruSeq® DNA PCR-Free Sample Preparation Kit (Illumina, USA) following the manufacturer’s recommendations with index codes added. Library quality was assessed on the Qubit@ 2.0 Fluorometer (Thermo Scientific) and Agilent Bioanalyzer 2100 system. The library was then sequenced on an Illumina HiSeq 2500 platform with 250 bp paired-end reads generated.

### 16S rRNA sequence analysis

Paired-end reads was assigned to samples based on their unique barcode and truncated by removing the barcode and primer sequences. Paired-end reads (raw tags) were merged using FLASH (Version 1.2.7). Quality filtering on the raw tags were performed under specific filtering conditions to obtain high-quality clean tags according to the QIIME2 software quality control process. Tags were compared with the Gold database using the UCHIME algorithm to detect chimeric sequences, which were then removed sequences analysis was performed using Uparse (Version 7.0.1001). Sequences with ≥97% similarity were assigned to the same OTU. For each representative sequence, the GreenGene Database was used, based on the RDP classifier (Version 2.2) algorithm, to annotate the taxonomic information. To study the phylogenetic relationships of the different OTUs, and differences in the dominant species in each group, a multiple sequence alignment was constructed using MUSCLE (Version 3.8.31). OTU abundance information was normalized to the number of sequences for the sample with the least sequences. α-diversity and β-diversity were performed based on the normalized data and calculated with QIIME then displayed with R (Version 2.15.3).

### Metagenomic sequence analysis

All samples were paired-end sequenced on the Illumina platform (insert size 350 bp, read length 150 bp) at Novogene Bioinformatics Technology Co., Ltd. After quality control, the Clean Data was blasted to the dog genome database with Bowtie (Version 2.2.4, parameters: --end-to-end, −-sensitive, −I 200, −X 400.) to filter reads that are of host origin. The set of high-quality reads was then used for further analysis.

Clean data was executed using SOAPdenovo2 (Version 2.04). Assembled Scaftigs were disassembled at N connections to generate Scaftigs without Ns. Clean Data from all samples were compared to each Scaffolds using Bowtie (Version 2.2.4) to identify PE reads not used. All reads not used in the forward step were combined and then used for mixed assembly with SOAPdenovo2 (Version 2.04). Scaftigs (continuous sequences within scaffolds) < 500 bp were filtered before statistical analysis of both the single and mixed assemblies. ORFs in the Scaftigs (≥ 500 bp) assembled from both the single and mixed assemblies were predicted using MetaGeneMark (prokaryotic GeneMark.hmm Version 2.10). A non-redundant gene catalogue was then constructed with CD-HIT (Version 4.5.8).

Clean Data for each sample was mapped to the initial gene catalogue using Bowtie (Version 2.2.4) to obtain the number of reads for each gene mapped in each sample. Only genes with ≥2 mapped reads were retained and used for the subsequently analysis. The abundance of a gene was calculated by counting the number of reads that aligned to the gene and normalized by the gene length.

To access the taxonomic assignments of genes, genes were aligned to the integrated functional database, for example, CAZy database, using DIAMOND (Version 0.9.9, blastp, −e 1e-5). For each sequence’s blast result, the best Blast Hit is used for subsequent analysis. Resistance Gene Identifier (RGI) software was used to align the Unigenes to the CARD database with default parameter settings and blastp e value≤1e-5.

## Data Availability

All data generated or analysed during the current study will be available from the corresponding author upon repository and the NCBI sequence reads archive (SRA) under accession number PRJNA702770.

## Abbreviations

ARGs: Antibiotic resistance genes
GHs: Glycosyl Hydrolases
KEGG: Kyoto Encyclopedia of Genes and Genomes
MGA: Maltase-glucoamylase
NMDS: Nursing Minimum Data Set
OTUs: Operational taxonomic units
VAs: Veterinary antibiotics
